# mHealth-Supported Hearing Health Training for Early Childhood Development Practitioners: An Intervention Study

**DOI:** 10.3390/ijerph192114228

**Published:** 2022-10-31

**Authors:** Divan du Plessis, Faheema Mahomed-Asmail, Talita le Roux, Marien Alet Graham, Tersia de Kock, Jeannie van der Linde, De Wet Swanepoel

**Affiliations:** 1 Department of Speech-Language Pathology and Audiology, University of Pretoria, Pretoria 0028, South Africa; 2 Virtual Hearing Lab, Collaborative Initiative between University of Colorado and the University of Pretoria, Aurora, CO 80309, USA; 3 Department of Science, Mathematics and Technology Education, University of Pretoria, Pretoria 0028, South Africa; 4 hearX Foundation, Pretoria 0081, South Africa; 5 Ear Science Institute Australia, Subiaco 6008, Australia

**Keywords:** mHealth, community-based, telecare, hearing health, education and training, early childhood development, intervention

## Abstract

(1) Hearing health training and promotion is a priority for early childhood development (ECD) practitioners, but training opportunities are limited, especially in low- and middle-income countries (LMIC). mHealth (mobile health) has the potential to deliver scalable ear and hearing training to ECD practitioners. (2) This study investigated the effect of an mHealth training intervention program for ECD practitioners to improve knowledge and perceptions of hearing health in young children. An experimental one-group, pre-post-test study included ECD practitioners working with children between birth and 6 years old across 31 neighbouring communities in the Western Cape Province, South Africa. Hearing health training was provided using WhatsApp messages that encompassed infographics and voice notes. Knowledge and perceptions regarding hearing and hearing-related problems in children were surveyed pre-training, directly post training, and 6 months post training. (3) ECD practitioners (*N* = 1012) between 17 and 71 years of age received the mHealth training program and completed both the pre-and post-training surveys. Overall, knowledge scores indicated a significant improvement from pre- to post training (*Z* = −22.49; *p* < 0.001). Six-month post-training knowledge scores were sustained. Content analysis of ECD practitioners’ application of the training information 6 months post training indicated improved awareness, practical application, better assistance for hearing problems, and widespread advocacy. (4) The mHealth training program supports improved knowledge and perceptions of ECD practitioners regarding hearing health for young children. With improved knowledge scores maintained 6 months post training, mHealth hearing health training is an effective intervention. An mHealth training program for ECD practitioners provides a scalable, low-cost intervention for primary and secondary prevention in childhood hearing loss, especially in LMICs.

## 1. Introduction

### 1.1. Hearing Loss and Early Childhood Development

Hearing loss (HL) is one of the most prevalent developmental disorders, with 34 million children estimated to have disabling HL globally, of which 95% reside in low- and middle-income countries (LMIC) [1,2,3,4]. Hearing loss identification in young children is delayed typically due to absent screening programs, with late identification having far-reaching consequences [4,5,6], including listening and communication difficulties, delays in speech and language development, delayed cognitive development, poor academic achievement, and adverse effects on social and emotional well-being [4,5,7,8,9,10,11,12]. Furthermore, an estimated 60% of childhood hearing loss [3] is preventable through the implementation of public health measures, including immunization, adequate maternal and childcare practices, early identification, and management of common ear conditions through systematic screenings [3]. Therefore, early detection of childhood HL is the key to effective management to support optimal outcomes [1,3,13].

Early childhood development (ECD) programs aim to provide services for optimal childhood development while ensuring general health. ECD centres, particularly in LMICs and low-socioeconomic-status (SES) communities, serve as front-line healthcare platforms for early detection, with ECD practitioners often acting as primary caregivers [14,15]. Therefore, ECD practitioners play a critical role in preventing and detecting developmental risks in young children, including hearing loss [14,16]. A study by Abraham and colleagues [17] investigated the feasibility of training teachers at day-care centres regarding primary ear care and observed an increase in average knowledge scores from 28.4% pre-training to 86% post training. The training was provided through in-person teaching methods, which included lectures, discussions, role-plays, clinical examinations, and practice sessions [17]. Scalable interventions for hearing health education and promotion could leverage innovative approaches such as mHealth (mobile health) to make ear and hearing health training for ECD practitioners more accessible. mHealth refers to the utilization of mobile phones, wireless devices, and digital assistants to improve healthcare delivery [18,19]. With the implementation of mHealth, barriers, including limited availability of hearing health professionals, transportation, time, expenses, and resource availability, can be addressed [20]. Utilizing mHealth to educate and promote hearing health can enhance the capabilities of ECD practitioners to substantially reduce the morbidity related to ear and health disorders [21,22].

### 1.2. mHealth Intervention Solutions

Innovative mHealth approaches are rapidly becoming important for public health care in LMICs, providing tools for awareness, health promotion campaigns, and services that are accessible and can scale rapidly [20,23,24,25,26,27,28]. In addition, the ubiquity of mobile phones and increased internet access have created an opportunity to optimize the implementation of mHealth services and potentially increase the efficiency of various service-delivery cadres [18,19,24]. mHealth applications have been adopted across multiple professions and adapted for the intent of use in clinical and non-clinical environments [29,30]. Text-message interventions’ significant benefits include reminders, alerts, education, motivation, and prevention [29,31]. Individualized motivating SMS text messages or reminders are one example of how digitization allows health promotion and preventive interventions to be customized and tailored to the requirements of people [32].

Hearing health training and promotion through mHealth has the potential to support preventative hearing healthcare services for young children from underserved populations, but there is a lack of evidence on hearing health promotion through mHealth [20,32]. A recent scoping review by Frisby and colleagues [18] on mHealth and hearing loss identified only three papers reporting hearing health promotion, all published recently in 2021. Employing mHealth to train personnel such as ECD practitioners who serve as front-line workers in vulnerable communities can promote and support primary and secondary prevention of childhood hearing loss. However, limited evidence exists regarding the effectiveness of mHealth training interventions for ECD practitioners on hearing health in young children [28]. Therefore, this study investigated the effect of an mHealth training program on ECD practitioners’ knowledge and perceptions of hearing in young children.

## 2. Materials and Methods

### 2.1. Study Design

An experimental one-group, pre-post-test design was employed to determine the effect of an mHealth-supported intervention program on ECD practitioners’ knowledge and perceptions of hearing health in children immediately after training and 6 months post training. In addition, a secondary qualitative component reported how ECD practitioners used the training information during a 6-month interval since completing the training.

### 2.2. Recruitment

#### Participants

The project included ECD centres and schools of low SES across 31 neighbouring communities in Cape Town and Paarl Valley within the Western Cape Province. ECD centres and schools included community-based facilities that provided education and nurturing care to children from birth to 6 years of age. ECD centres and schools were selected based on previous hearing screening services provided by the hearX Foundation and Carel du Toit Centre and Trust. Additionally, new ECD centres and schools were identified and approached, and a referral-based method was utilized allowing participating ECD practitioners to refer new practitioners. The principals of selected ECD centres and schools were contacted to obtain the contact details of willing participants. Participants included ECD practitioners working with young children between birth and 6 years of age at ECD centres and schools. For this study, the term *ECD practitioners* was used to encompass various positions at an ECD centre or school, including principals, teachers, assistants, volunteers, administrators, mentors, or field workers. ECD centres and schools essentially provide similar services ensuring quality early-childhood development, with teachers and professionals receiving equivalent ECD training (Multimedia Supplementary S1).

Inclusion criteria for participants included self-reported proficiency in English and access to WhatsApp and mobile data on their phones. Participants also had different levels of educational training in terms of ECD levels (Multimedia Supplementary S1). Participants were contacted telephonically by representatives of the *3E project* (Ears and Eyes for Education) facilitated by local Non-Governmental Organizations (NGOs) (hearX Foundation and Carel du Toit Centre and Trust) in the selected communities within the Western Cape Province. The *3E project* representatives were lay community health workers trained in providing hearing screenings. The *3E project* representatives were allocated to the specific district parts of the selected communities and assigned to remotely communicate and facilitate training to the ECD practitioners for the specific ECD centres over WhatsApp. Several ECD centres were previously involved in school hearing screening services provided by the hearX Foundation prior to the commencement of this study. This paper reports data from participants who received ear and hearing health training through the mHealth training program between June 2020 and July 2021.

### 2.3. mHealth Hearing Training Program

The training program’s goal was to equip ECD practitioners with the necessary knowledge and practical application of information to actively participate in the process of identifying and supporting children with potential hearing difficulties and where necessary make appropriate referrals. The *3E project* used the EARS (Early, Academics, Red flags, and Support) training program (Multimedia Supplementary S2) for ECD practitioners to achieve its goal. The training program guided ECD practitioners through critical concepts related to hearing loss in young children during the developmental ECD period, the importance of healthy hearing for healthy learning, and appropriate risks that warrant a referral.

#### 2.3.1. Development of mHealth Training Material

Training material was developed through a collaborative initiative by the hearX Foundation, Carel du Toit Centre and Trust with the University of Pretoria. The four-day training material consisted of a daily pre-recorded voice note accompanied by an infographic (Multimedia Supplementary S2) pertaining to each day’s ascribed topic (e.g., day 1–early). Each day’s voice note included detailed educational information focusing on theoretical content and practical application that ECD practitioners could apply in and outside ECD centres or schools. The accompanied infographic concisely displayed the most important information from the daily voice note. The voice note and infographic were in English and designed specifically for distribution over WhatsApp via instant messages. A readability consensus was applied to evaluate the readability of the information provided over the four-day training program. The information was processed through seven readability formulas (Flesch Reading Ease score, Gunning Fog, Flesch–Kincaid Grade Level, The Coleman–Liau Index, The SMOG Index, Automated Readability Index, and Linsear Write Formula), and the readability consensus score was at fifth- to sixth-grade reading level, which is classified as fairly easy to read. The infographic provided ECD practitioners with a concise display of important information on that day’s topic with the intent to listen to the voice note while viewing the infographic simultaneously. The content for each day’s training covered essential intervention aspects and learning objectives relating to healthy hearing in young children (Table 1) and the importance of early identification of a hearing problem by observing a child’s behaviour and possible red flags relating to a hearing problem. Additionally, it covered how a hearing problem can affect a child’s academic achievement if the necessary support is not received.

#### 2.3.2. Presentation of Training Material

All communications, training, and feedback were conducted via WhatsApp (version 2.21.11). Participants were telephonically introduced to the program on a Friday, and upon acceptance to participate, they received a detailed explanation of the program and the pre-training survey. Training days only occurred on weekdays, which totalled four days of training. Participants were informed that training would commence on a Monday. After receiving the participants’ completed pre-training survey, the allocated 3E representatives sent the respective voice note and daily infographic over the four remaining training days. Participants could listen to the voice notes as often as they preferred while viewing the infographic. The voice notes and daily infographics are stored in the WhatsApp chat between the 3E representative and ECD practitioner even after the completion of the training program. After completing the training program, participants received a certificate of completion via WhatsApp.

#### 2.3.3. Data Collection Material

ECD practitioners’ knowledge and perceptions regarding hearing in ECD and hearing-related problems in children were surveyed pre-training, directly post training, and 6 months post training. The survey was adapted based on work from van Wyk and colleagues [33]. All three surveys consisted of the same 12 closed-ended items (Multimedia Supplementary S3) pertaining to the learning objectives specified for the training material. Participants were asked to rate each of the 12 items on a 5-point Likert scale ranging from (1) strongly disagree to (5) strongly agree. Participants’ scores were summed from the 12 survey items, and a knowledge score was obtained per participant and as a collective group. The knowledge scores were utilized to compare the changes in scores over the three surveys. In addition to these 12 items, the post-training survey also included two additional closed-ended 5-point Likert scale items allowing participants to rate the training received. The 6-month post-training survey included one additional closed-ended 5-point Likert scale item and an open-ended feedback question probing participants’ practical application of the training information.

Moreover, the 6-month post-training survey also evaluated the generalization of the training program after 6 months. The surveys were compiled in English and made available in two formats: an online Google form (sent as a WhatsApp link) or a WhatsApp message. Participants who indicated to their 3E representative that they could not access the Google form due to limited available mobile data received the survey in a WhatsApp message format.

### 2.4. Procedures

The principal of each ECD centre or school was contacted telephonically, received all ethical disclosures, and was introduced to the EARS training program. Principals could accept or reject on behalf of their ECD centre or school to partake in the EARS training program. Principals were requested to speak to their staff and obtain permission to share the contact details of those interested in participating in the training program. On condition of acceptance, ECD practitioners were contacted telephonically by the 3E representatives, introduced to the EARS’ training program, and invited to participate. The telephonic explanation included what the EARS training program entailed and the topic of *the importance of healthy ears and hearing for healthy learning*, along with what participants could expect over the four days after accepting to participate in the program. The ECD practitioners were informed that they would receive a certificate of completion after the EARS training program and completed three surveys. ECD practitioners would also be placed into a lucky draw to be 1 of 10 participants to receive a monetary incentive, including a voucher of ZAR 250 (USD 17.50) at specified supermarkets. Before the ECD practitioners started training, they either received a WhatsApp message from the 3E representative with a link directing them to the online pre-training survey or a WhatsApp message containing the survey itself. ECD practitioners received a participation number for all documentation purposes and were required to use the same participant number throughout the training program.

Each day, participants received the mHealth training material through WhatsApp. After completing the four-day training program, all participants received a congratulatory message for completing the program and were notified to complete the post-training survey by either a link to the online Google form or the WhatsApp message containing the post-training survey.

**Figure 1:**
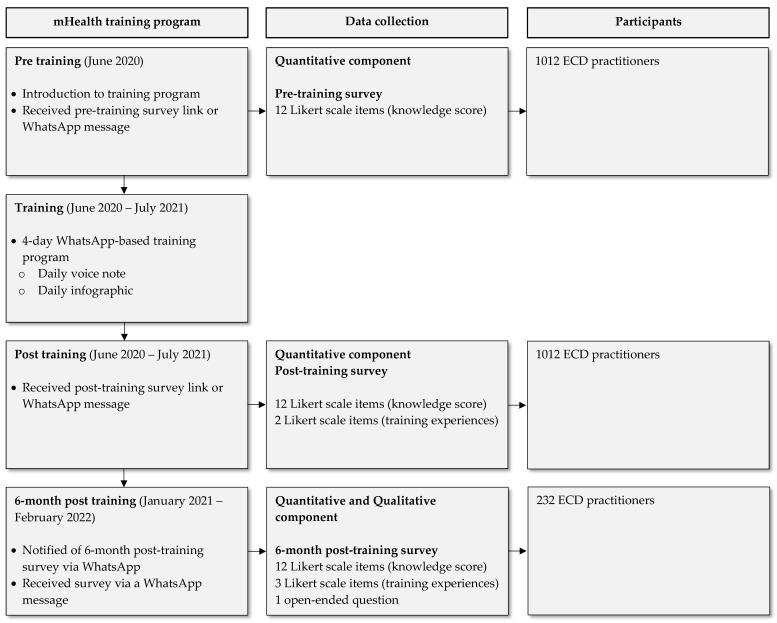
Data collection procedures linked to the mHealth training program.

A WhatsApp message informed participants about the 6-month post-training survey. Participants willing to complete the 6-month post-training survey were included in another lucky draw to be one of five participants to receive a monetary incentive, including a voucher of ZAR 350 (USD 23.50) at specified supermarkets. Data collection procedures linked to the mHealth training program are outlined in Figure 1.

### 2.5. Statistical Analysis

#### 2.5.1. Quantitative Analysis

Data were analysed using the Statistical Package for the Social Sciences (SPSS) version 27 (International Business Machines (IBM) Corp., 2020) except for the power analysis, which was conducted using G*Power version 3.1.9.4 [34]. For the achieved power analysis, a conservative approach was used where the smallest sample sizes were used. Accordingly, for the Kruskal–Wallis (*H*) test and for the Wilcoxon signed-rank (*Z*) tests, which determine whether differences between unrelated and related groups are statistically significant, respectively, the 6-month post-training group (*n* = 232) was used, and for the Kruskal–Wallis post hoc test, the smallest sample sizes concerning pairwise comparison was used (*degree in education and ECD level 6* (*n*_1_ = 33) and *ECD level 1 to 3* (*n*_2_ = 35)). For Spearman correlations (*r_s_*), used to test relationships between variables, the 6-month post-training group (*n* = 232) was used. For all power analyses, a medium to large effect was considered [35], and a 0.05 level of significance was used. For these tests, Kruskal–Wallis, Kruskal–Wallis post hoc, the Wilcoxon signed-rank test and the Spearman correlation, the achieved power was 0.864, 0.851, 1.000 and 1.000, respectively, indicating that these tests are 86.4%, 85.1%, 100.0% and 100.0% strong, and when comparing larger samples sizes, a more powerful analysis was obtained, which yields higher statistical values. Inferential statistical analysis was used to determine the strength and relationship between variables and observed change.

Descriptive statistical measures were used to quantitively analyse demographic information of the study sample along with determining the mean and standard deviations of the knowledge scores for each survey, respectively. A higher knowledge score (5) indicated better knowledge, and a lower score (1) indicated poorer knowledge. For the continuous variables, normality was tested using the Shapiro–Wilk test, and since all the *p*-values were less than 0.05, the data differed significantly from normality [36]. Accordingly, nonparametric methods were used. Inferential statistics included conducting the Kruskal–Wallis test to determine whether there were significant differences in survey scores between different independent variable groups, language, position at the ECD centre, and level of training. If a statistical significance (*p* < 0.05) was found between the categories of a variable, a subsequent post hoc pairwise comparison was performed using Dunn (1964) procedure. Spearman correlations was used to test for associations between variables. Stepwise linear regression models were built with predictors, participant age (continuous variable), previously exposed to screening procedures (binary variable with “*no”* as benchmark), participant home language (three categories with *English* as benchmark), level of training (four categories with *no formal training and other* as benchmark), and the position at an ECD centre (three categories with *assistant/volunteer* as benchmark). For the regression models, the recommendation of at least ten observations per predictor was met [37], assuring that the regression models were statistically powerful.

#### 2.5.2. Qualitative Analysis

The open-ended question in the 6-month post-training survey was qualitatively analysed using content analysis. A direct content analysis approach was followed by utilizing an open-ended question probing to investigate participants’ experiences and application of the EARS training information 6 months after the training. The content analysis involved categorizing statements into meaningful units that collectively described participants’ statements, representing ideas or topics of interest. Categorization concluded with organization into main categories with sub-categories along with the frequency distributions in a tabulated format.

## 3. Results

### 3.1. Demographic Characteristics of Study Sample

A total of 1012 ECD practitioners between 17 and 71 years of age (mean 37.67, SD 10.78) were included in the pre- and post-training of the study, and 232 of those between 19 and 65 years of age (mean 36.97, SD 10.49) also completed the 6-month post-training survey (Table 2).

### 3.2. Pre-Training Knowledge Scores

Table 3 provides a summary of the pre-and post-training knowledge scores. There was a significant difference across the average overall pre-training knowledge score for different languages (*H*(2) = 94.79, *p* < 0.001) and levels of training groups (*H*(3) = 48.54, *p* < 0.001). Note that, for language, for all inferential statistics, the category “*other”* was not considered, as it only applied to 1.1% of the participants. For the language groups, post hoc analysis indicated that both *English* (mean 4.01, SD 0.36) and *Afrikaans* (mean 4.01, SD 0.34) groups scored significantly higher than the *isiXhosa* group (mean 3.74, SD 0.44). The level of the training groups’ post hoc analysis revealed significant differences in pre-training scores between all levels of training except between the *no formal training and other* and *degree in education and ECD level 6*. ECD practitioners with *no formal training and other* drew attention upon analysis because the group had significantly higher pre-training knowledge scores (mean 3.94, SD 0.38) compared to ECD practitioners with *ECD level 1 to 3* (mean 3.71, SD 0.49) and *ECD level 4 to 5* educational training (mean 3.81, SD 0.43).

A stepwise linear regression model (Table 4) was statistically significant (*F* [6, 994] = 27.69, *p* < 0.001), and predictors explained 14.3% of the variation in overall pre-training survey scores. Older participants (*β* = 0.05, *p* = 0.001) and previous exposure to hearing screenings (*β* = 1.20, *p* < 0.001) significantly contributed to higher pre-training scores (Table 4). For the latter, participants exposed to hearing screenings had a significantly higher overall knowledge score than participants who were not exposed to hearing screenings previously. Four predictors were associated with significantly lower pre-training knowledge scores (Table 4), namely *isiXhosa* as a home language (*β* = −3.31, *p* < 0.001) when benchmarked against *English*, *ECD training levels 1 to 3* (*β* = −2.19, *p* < 0.001) and *ECD training levels 4 and 5* (*β* = −1.19, *p* < 0.001) when benchmarked against *no formal training or other*, and participants who are *principals* (*β* = –0.89, *p* = 0.03) when benchmarked against *assistants/volunteers*.

### 3.3. Post-Training Knowledge Scores

The post-training mean score was significantly (*Z* = −22.49, *p* < 0.001) higher than the pre-training mean score across each of the 12 survey items (Multimedia Supplementary S3; *p* < 0.001; Table 3). Increasing age was negatively associated with the overall improvement in post-training knowledge scores (*r_s_* = −0.11, *p* < 0.001). Language groups demonstrated significantly different overall improvements from pre- to post-training scores (*H*(2) = 44.89, *p* < 0.001). Participants speaking *isiXhosa* had the highest mean improvement of (mean 0.59, SD 0.60) from the pre- to the post-training survey, and participants speaking *Afrikaans* had the lowest mean improvement score of (mean 0.32, SD 0.38). Post hoc tests revealed significant pairwise differences between all pairwise language comparisons except between the *English* and *Afrikaans* groups for improvement scores, indicating that the two groups had similar improvement knowledge scores after the training. 

Level of training demonstrated different overall improvements from pre- to post-training scores (*H*(3) = 12.98, *p* = 0.005). Participants with *ECD training levels 1 to 3* had the highest mean improvement score (mean 0.55, SD 0.60), while participants with *no formal training and other* had the lowest mean improvement knowledge score of (mean 0.38, SD 0.47). Post hoc testing revealed significant pairwise differences between *degree in education and ECD level 6* (mean 0.40, SD 0.52) and *ECD level 4 and 5* (mean 0.52, SD 0.55), between *no formal training and other* (mean 0.38, SD 0.47) and *ECD levels 1 to 3* (mean 0.55, SD 0.60), and between *no formal training and other* (mean 0.38, SD 0.47) and *ECD level 4 and 5* (mean 0.52, SD 0.55). With the *ECD levels 1 to 3* participants showing the highest improvement, it can be argued that this group could have felt that the training would be beneficial for them and put in their best efforts to learn from the training, which clearly paid off.

A stepwise linear regression model (Table 4) on predictors for improvement in knowledge scores significantly explained 7.1% of the variation (*F* [3, 997] = 25.52, *p* < 0.001). Participants’ home language and position at ECD centres or schools significantly contributed to improved knowledge scores. For the latter, participants in the *principal* position had a significant improvement (*β* = 1.69, *p* = 0.002) in their overall knowledge scores when compared to participants in the *assistant/volunteer* position (Table 4). For the former, participants with *isiXhosa* as a home language showed a significant increase (*β* = 2.82, *p* < 0.001) in their overall improvement knowledge scores when compared to participants with *English* as a home language (Table 4). Participant age was significantly associated (*β* = −0.10, *p* < 0.001) with mean improvement scores in that for every one year older, the score decreased by 0.10 on average.

In addition to the 12 items in the post-training survey, participants were asked if the information provided during the training program was meaningful. The majority of participants (770/1012, 76.1%) either strongly agreed or agreed (227/1012, 22.4%) that the training was meaningful to them. Additionally, participants were asked whether the training increased their knowledge of hearing problems in young children. Most participants (750/1012, 74.1%) strongly agreed that the training increased their knowledge, while others (250/1012, 24.7%) agreed.

### 3.4. Six-Month Post-Training Knowledge Scores

The 6-month post-training mean knowledge score (mean 4.38, SD 0.35) was significantly higher than the pre-training mean knowledge score (*Z* = −11.36, *p* < 0.001) but did not differ significantly from the post-training mean knowledge score (*Z* = −0.45, *p* = 0.65). Based on the Wilcoxon signed-rank test, across all 12 items, there were no significant differences between the post-training knowledge scores and 6-month post-training knowledge scores except for item 1 (*Z* = −2.47, *p* = 0.01) and item 11 (*Z* = −2.04, *p* = 0.04). There was a significant mean difference in knowledge scores between the three participant position groups at ECD centres (*H*(2) = 11.09, *p* = 0.004), with the *assistant/volunteer* group having the highest mean score of (mean 4.48, SD 0.33) and the *principals* having the lowest mean score of (mean 4.26, SD 0.33). A post hoc test revealed significant pairwise differences between all pairwise comparison position groups except between *teachers* and *assistants/volunteers*.

### 3.5. Six-Month Post-Training Content Analysis

Table 5 provides the categories and sub-categories identified for 181 ECD practitioners’ responses to the open-ended question in the 6-month post-training survey. The question prompted ECD practitioners to indicate any way that they used the information received during the EARS training program during the 6-month interval since completing the online training.

## 4. Discussion

The mHealth hearing training program improved ECD practitioners’ knowledge scores significantly, with improvements maintained at 6 months post training. Almost all (997/1012, 98.5%) of the ECD practitioners experienced the training to be meaningful and reported improved knowledge of hearing health in young children. A concise multimedia mHealth hearing training program is a low-cost, scalable intervention to equip ECD practitioners with knowledge to identify and refer at-risk children and to support children with hearing difficulties in the class environment.

ECD practitioners with the highest level of training (*degree in education and ECD level 6*) had significantly higher pre-training knowledge scores than practitioners with the lowest level of training (*ECD level 1 to 3*). Those ECD practitioners with the lowest level of training demonstrated the largest improvement in post-training scores. Improved knowledge scores confirm that the mHealth training program provided meaningful educational information and that the training successfully met learning objectives on hearing health in young children (Table 1). ECD practitioners’ knowledge is crucial for inclusive ECD classes, where exceptional teaching and caring practices are necessary for the holistic development of vulnerable children [14,38]. With the widespread diversity and background of children in ECD centres and schools, especially in LMICs, teachers and ECD practitioners need specialised knowledge competencies and skills to provide nurturing care and an auspicious learning environment for children with special needs [38].

ECD practitioners’ demographic characteristics had varying effects on their pre-training knowledge scores. Significantly higher pre-training knowledge scores were evident for ECD practitioners previously exposed to hearing screenings in their facilities. This emphasises an important additional benefit of hearing screening in ECD centres and schools, resulting in improved general knowledge of hearing health in children. Home language and English proficiency were also important with *isiXhosa*-home-language ECD practitioners having lower knowledge scores in the pre-training survey when compared to ECD practitioners with *English* as their home language. Interestingly, ECD practitioners speaking *isiXhosa* showed the highest mean knowledge improvement in the post-training survey. The information provided in the voice notes and daily images were fairly easy to understand, with readability consensus at a fifth- to sixth-grade reading level ensuring accessibility of the content. The 6-month post-training knowledge scores remained consistent, demonstrating that the level of information furthermore supported long-term retention. Future training should, however, be explored in versions that support different home languages to facilitate wider accessibility.

Additional improvements in post-training knowledge were related to age and position in ECD centres and schools. Linear regression indicated that ECD practitioners working as a *principal* at an ECD centre or school had a lower knowledge score in the pre-training survey when compared to *assistants/volunteers*. Younger ECD practitioners are typically perceived as knowledge receivers [39]. A study by Burmeister and colleagues [39] investigated the effect of age on the knowledge transfer process, indicating younger participants were more able and motivated than older participants to receive knowledge in the knowledge transfer process. Therefore, younger participants seemed to assimilate the educational material with greater ease leading to higher improvement scores. Campbell-Barr [40] stated that ECD knowledge encompasses more than just theoretical knowledge. The Bernstein model on knowledge acquisition through practice acknowledged that professionals in early development, such as ECD practitioners, utilize both theoretical and practical knowledge [40]. Therefore, practical experience is an important factor that influences the knowledge of ECD practitioners, as was also observed with higher pre-training knowledge scores for ECD facilitators with prior hearing screening exposure. It is likely that *principals* at the ECD centres or schools are less involved in everyday classroom activities and educational settings, which could have contributed to lower pre-training knowledge scores. The *principals* did, however, demonstrate significant improvements in post-training knowledge scores.

Content analysis analysed the ways in which ECD practitioners used the training material after 6 months post mHealth training. The analysis identified various categories and sub-categories, including improved awareness, practical application, better assistance for hearing problems, and widespread advocacy. Two prominent categories included *identifying hearing problems in children* and *sharing information*. Almost half (77/181, 42.5%) of the ECD practitioners reported *identifying hearing problems* better and in the sub-category (51/77, 66.2%) of the ECD practitioners reported being *more observant and aware of hearing problems*. More than a quarter (50/181, 27.6%) of ECD practitioners indicated they shared the training information with colleagues, parents, and community members. The training improved ECD practitioners’ competence and supported confidence in approaching young children with hearing problems and applying training information in the classroom setting. For example, one ECD practitioner indicated they *will be able to take care of a child with any hearing problems*, and another practitioner mentioned that they *could adapt lessons and assessments to meet children’s needs*.

The mHealth training program for ECD practitioners provides a scalable, low-cost training for primary and secondary prevention of childhood hearing loss, especially in low-and middle-income countries and communities of low SES. Furthermore, the provision of training material via an instant-messaging application has important advantages, including cost-effective administration of an intervention program, efficient management, and easy distribution across geographic boundaries [28,41,42]. This study was the first of its kind to evaluate the use of an mHealth training program to support knowledge and awareness of hearing health [20]. An important strength of this study was its ecological validity, with broad inclusion criteria representative of community ECD centres and schools in low-income settings.

Further investigation warrants implementing culturally tailored resources and mHealth intervention and training for ECD practitioners in their home languages. Moreover, evaluation of the impact of an mHealth training program on the identification of children with hearing problems could be investigated.

### Study Limitations

The study had a large sample size (*n* = 1012), with participants in both the pre- and post-surveys. However, a limitation of this study was a low response rate for the 6-month follow-up survey, with approximately only one-quarter (232/1012, 22.9%) of the participants from the original sample completing the follow-up survey. The low response rate may partly have been due to survey fatigue [43]. Participants used their mobile phones and cellular data to complete the mobile-based survey. The cost of cellular data could have prohibited participants from accessing the WhatsApp-based survey. Additionally, participants may have changed mobile numbers during the 6-month interval and therefore did not receive the 6-month follow-up survey. Furthermore, large sample sizes like those in this study can result in detection of small effect sizes with high power. This means a result may be flagged as statistically significant when it does not have real-world significance [44]. While this may be a potential limitation of this study, the consistent pattern of significant improvement across all pre- and post-questions and no significant differences between immediate post and 6-month post questions suggest that this was not the case.

## 5. Conclusions

ECD practitioners’ knowledge and perceptions regarding hearing health in young children are critical for primary and secondary prevention in childhood hearing loss, especially in countries with low SES, where ECD facilities may serve as the initial point of contact for hearing screening. An mHealth intervention program can support improved knowledge and perceptions of ECD practitioners regarding hearing health for young children, with generalization maintained over time. Employing an mHealth training program provides a scalable, low-cost intervention for prevention in childhood hearing loss, especially in low- and middle-income countries.

## Figures and Tables

**Table 1:** Training description and learning objectives with survey items.

Training Components	Learning Objectives	Items from Survey ^1,2^ (Multimedia Supplementary S3)
Part 1—“E” for Early Importance of early identification of hearing problems and early intervention. *Duration ^3^: 2 min, 12 s*	To understand the importance of identifying a hearing problem as soon as possible in children. To understand the importance of ear health in children.	Items 2, 5, and 6
Part 2—“A” for Academics Influence of hearing problems on academic performance and importance of healthy hearing for healthy learning. *Duration ^3^: 2 min, 35 s*	To describe why healthy hearing is important for healthy learning. To understand how and why hearing problems result in poor school progress.	Items 8, 9, and 10
Part 3—“R” for Red Flags Possible red flags to look out for and how this can be indicative of hearing problems in children. *Duration ^3^: 2 min, 40 s*	To describe the different types of hearing problems. To understand and identify the red flags (warning signs) of hearing problems in children.	Items 3, 4, and 7
Part 4—“S” for Support Ways teachers can support children with potential or diagnosed hearing problems. *Duration ^3^: 2 min, 50 s*	To understand the referral process of a child with hearing problems to an audiologist. To understand how to support the child with a hearing problem in the classroom.	Items 6, 10, 11, and 12

^1^ 5-point Likert scale used: (1) strongly disagree, (2) disagree, (3) neutral, (4) agree, and (5) strongly agree. ^2^ Twelve items linked to the EARS training content that were used to determine knowledge scores. ^3^ Duration of the daily voice note.

**Table 2:** Demographic characteristics of participants completing the pre-training, post-training (*n* = 1012), and 6-month post-training (*n* = 232) survey.

	Pre- and Post-Training Survey (*n* = 1012)	Six-Month Post-Training Survey (*n* = 232)
	*n* (%)	*n* (%)
Gender		
*Female*	958 (94.7)	222 (95.7)
*Male*	54 (5.3)	10 (4.3)
Home language		
*isiXhosa*	571 (56.4)	135 (58.2)
*English*	221 (21.8)	44 (19.0)
*Afrikaans*	209 (20.7)	52 (22.4)
*Other*	11 (1.1)	1 (0.4)
Position at ECD centre or school		
*Principal*	219 (21.6)	53 (22.8)
*Teacher*	607 (60.0)	128 (55.2)
*Assistant or Volunteer*	186 (18.4)	51 (22.0)
Level of training		
*Education degree (B.Ed.) and ECD level 6*	159 (15.7)	33 (14.2)
*ECD level 4 and 5*	537 (53.1)	125 (53.9)
*ECD level 1 to 3*	130 (12.8)	35 (15.1)
*No formal training and other*	186 (18.4)	39 (16.8)
Previous exposure to hearing screening		
*Yes*	580 (57.3)	140 (60.4)
*No*	432 (42.7)	92 (39.7)

**Table 3:** Descriptive statistics, the *z*-test statistic and the corresponding *p*-values of the pre-post test scores of ECD practitioners (*n* = 1012).

Survey Items	Pre-Training Mean Score (SD) ^1^	Post-Training Mean Score (SD) ^1^	Mean Improvement (SD)	*Z*	*p*-Value
ECD staff and teachers know enough about hearing and hearing problems in children.	2.77 (1.06)	3.43 (1.26)	0.66 (1.39)	−13.81	<0.001
2. A child can be born with hearing problem.	4.15 (0.64)	4.53 (0.58)	0.38 (0.79)	−14.27	<0.001
3. There are different types of hearing problems.	3.89 (0.85)	4.50 (0.61)	0.61 (0.90)	−18.26	<0.001
4. Ear infections can cause hearing problems.	4.04 (0.74)	4.60 (0.57)	0.56 (0.86)	−17.57	<0.001
5. It is important to know if a child has hearing problems at an early age to help them.	4.37 (0.67)	4.71 (0.52)	0.34 (0.75)	−13.51	<0.001
6. Hearing problems in young children cannot be treated. ^2^	3.56 (1.00)	4.05 (1.04)	0.49 (1.33)	−11.14	<0.001
7. There are signs in a child’s behaviour that may tell you if the child has a hearing problem.	4.10 (0.68)	4.58 (0.57)	0.48 (0.82)	−16.20	<0.001
8. Hearing problems can make learning to read and write difficult.	3.99 (0.84)	4.50 (0.72)	0.51 (0.87)	−16.20	<0.001
9. Hearing problems can make concentration in a classroom difficult.	4.24 (0.62)	4.58 (0.62)	0.34 (0.78)	−13.04	<0.001
10. Even with treatment, children with hearing loss cannot achieve the same as other children in school. ^2^	3.16 (1.10)	3.58 (1.30)	0.42 (1.40)	−8.83	<0.001
11. If someone thinks a child has a hearing problem, the child should be sent to an audiologist.	4.14 (0.61)	4.61 (0.56)	0.47 (0.71)	−17.84	<0.001
12. A child with a hearing problem can hear better in school if they sit in the front row of the classroom.	3.83 (0.83)	4.38 (0.82)	0.55 (0.97)	−16.01	<0.001
**Overall mean**	3.85 (0.43)	4.34 (0.41)	0.48 (0.54)	−22.49	<0.001
**Total**	46.25 (5.12)	52.04 (4.91)	5.79 (6.48)		

^1^ Likert response category ranged from (1) strongly disagree, (2) disagree, (3) neutral, (4) agree, and (5) strongly agree. ^2^ Likert scale score inverted based on negative stated item.

**Table 4:** Linear regression analysis results for pre-training and improvement knowledge scores.

Model	*R* ^2 1^	Unstandardised Coefficients		Standardised Coefficients	*T*	*p*-Value	95.0% Confidence Interval for *β*	
		*β* ^2^	Std. Error	*β*			Lower Bound	Upper Bound
Pre-training	0.143	46.59	0.63		73.68	<0.001	45.35	47.83
*Age*		0.05	0.02	0.11	3.33	0.001	0.02	0.08
*Previously exposed to screening procedures*		1.20	0.33	0.12	3.68	<0.001	0.56	1.84
*Home language—isiXhosa*		−3.31	0.33	−0.32	−9.89	<0.001	−3.96	−2.65
*Level of training—ECD level 1 to 3*		−2.19	0.51	−0.14	−4.35	<0.001	−3.18	−1.20
*Level of training—ECD level 4 and 5*		−1.90	0.34	−0.12	−3.45	<0.001	−1.87	−0.51
*Participants’ work position—Principal*		−0.89	0.42	−0.07	−2.14	0.03	−1.71	−0.08
Improvement knowledge scores	0.071	7.47	0.79		9.41	<0.001	5.91	9.03
*Age*		−0.10	0.02	−0.16	−4.71	<0.001	−0.14	−0.06
*Home language—isiXhosa*		2.82	0.40	0.22	7.06	<0.001	2.04	3.61
*Participants’ work position—Principal*		1.69	0.54	0.11	3.16	0.002	0.64	2.74

^1^ Determination coefficient for proportion of variance in the knowledge scores (dependent variable) predicted by the independent variables. ^2^ Effect on the overall knowledge score (dependent variable) per model.

**Table 5:** Application of the EARS training information reported 6-month post training by ECD practitioners and analysed qualitatively (*n* = 181).

**Categories**	**Sub-Categories**	**Illustrative Responses from ECD Practitioners**
Identifying hearing problems in children (*n* = 77)	Notice and refer children with hearing problems	*“I have identified many children in my school and community, it helps a lot.”* *“The training helped me because there is one child that I was suspecting, and it turns out that I was right.”* *“Refer children who I suspect that they have hearing problem to audiologists where they get checked”.*
	More observant and aware of hearing problems	*“I’ve learned a lot because I can see if the child is not hearing before someone’s tells me.”* *“Yes, it helped me out a lot and now I know how to identify or see a child who can’t hear properly.”* *“I started to observe my children to see if anyone of them has a hearing problem.”* *“I start looking at behaviours of children to see if I pick up any signs that they might have a hearing problem.”*
Sharing information (*n* = 50)	Share information with colleagues	*“I have shared the important information with the ECD teachers whom I am working with in the playgroup sessions.”* *“Shared my knowledge with my ECD principals.”* *“I have printed out all the slides and put it in a file and I have made copies and shared the information with colleagues and primary school teachers.”*
	Share information with parents	*“I gained lots of knowledge of hearing problems in children and share it with the parents and the community and they must look out for symptoms in the child and what to do if the has a hearing problem and where to go.”* *“I address the information in our parents WhatsApp group.”*
	Share information with community members	*“I educate the community about the hearing loss in children.”* *“I managed to share the whole information with the community and my neighbours.”*
Apply information in the classroom setting (*n* = 27)		*“I was more aware and could adapt my lessons and assessments to meet children’s needs.”* *“I am always watching in the classroom if every child is in the right place that he/she can hear me.”* *“I now look at the children when I’m teaching, and let them seat in front row, so that they can read my lips. I always use different learning styles—visual learning style to accommodate children who cannot hear.”* *“I will let her sit in front and make sure I position myself in the ear that she can hear me from…”*
Assisting children with hearing problems (*n* = 21)		*“I am more informed now and will be able to take care of a child with any hearing problems.”* *“Strategies to help her was put in place, she’s doing very well with eye contact and in her academics.* *”* *“I am going more down to the child’s level to look straight in the face when I’m talking to the child.”*
Assist and advise parents of children with hearing problems (*n* = 6)		*“I can… advise parents better regarding certain problems their child has.”* *“I can… provide parents with needed help.”* *“I can also advise a parent what to do with the child and the best way to handle the problem and where to take the child who have ear infections/problem.”*

## Data Availability

The authors can be contacted to access the data.

## Supplementary Materials

The following supporting information can be downloaded at: https://www.mdpi.com/article/10.3390/ijerph192114228/s1.
