# Supplementary material for: mHealth-Supported Hearing Health Training for Early Childhood Development Practitioners: An Intervention Study
Source: Int J Environ Res Public Health. 2022 Oct 31;19(21):14228. doi: 10.3390/ijerph192114228 (PMC9658621; doi:10.3390/ijerph192114228)
Supplement: Supplementary file 1 [file ijerph-19-14228-s001.zip › Multimedia Supplementary S1/Multimedia Supplementary S1.pdf]

Table 1. Level of educational training within different ECD levels of qualifications.

**B. Ed. Degree and ECD level 6**<br>Individuals of professional competence informed by sound knowledge and understanding of their area of specialization. The B.Ed. degree is an initial teaching qualification for candidates to be registered as fully qualified professional educators in schooling. The primary purpose of the ECD level 6 or ACE is to provide for training in a new phase or subject specialization, such as: textbook writing, computers in education, assessment, and quality assurance in education.
**ECD level 5 and ECD level 4**<br>To enable educators to create and administer a learning program based on their understanding of child development from birth to age nine. To strengthen community based ECD services for young children by improving teaching skills and/or offering chances for support and leadership training. Develop ECD educators with a strong practical competence to offer high-quality early childhood development services in community-based settings for children ages 2 to 6. Allow students to assist others.
**ECD level 1 to ECD level 3**<br>Learners have the requisite knowledge, skills, and attitudes to deal with the difficulty of incorporating children with learning and developmental disabilities in ECD settings. People who meet this Unit Standard can plan and prepare for interactions with infants, toddlers, and/or young children. They can also engage in a range of interactions with the children with whom they work. Enable learners to recognize and react to the basic requirements of young children in all areas of their development throughout a given developmental stage and in a specific context, such as a center or at home.
