# Supplementary material for: mHealth-Supported Hearing Health Training for Early Childhood Development Practitioners: An Intervention Study
Source: Int J Environ Res Public Health. 2022 Oct 31;19(21):14228. doi: 10.3390/ijerph192114228 (PMC9658621; doi:10.3390/ijerph192114228)
Supplement: Supplementary file 1 [file ijerph-19-14228-s001.zip › Multimedia Supplementary S3/Multimedia Supplementary 3.pdf]

Table 2. Surveys on ECD practitioners' knowledge and perceptions of hearing related ECD in young children.

Pre-training survey[1, 2]

1. ECD staff and teachers know enough about hearing and hearing problems in children.
2. A child can be born with hearing problem.
3. There are different types of hearing problems.
4. Ear infections can cause hearing problems.
5. It is important to know if a child has hearing problems at an early age to help them.
6. Hearing problems in young children cannot be treated.[3]
7. There are signs in a child's behavior that may tell you if the child has a hearing problem.
8. Hearing problems can make learning to read and write difficult.
9. Hearing problems can make concentration in a classroom difficult.
10. Even with treatment children with hearing loss cannot achieve the same as other children in school.[3]
11. If someone thinks a child has a hearing problem the child should be sent to an audiologist.
12. A child with a hearing problem can hear better in school if they sit in the front row of the classroom.

Post-training survey [1, 4]

13. The information provided during the training program was meaningful to me.
14. The training program improved my knowledge on hearing problems in young children.

Six-month post-training survey [1, 5]

15. After completing the EARS training, I am more aware of hearing problems in children than before.
16. Can you tell us of any way that you have used the information you received during the EARS Training?

[1] Response categories ranged from (1) strongly disagree, (2) disagree, (3) neutral, (4) agree, (5) strongly agree. [2] 12 Likert scale Items used to determine ECD practitioners' knowledge scores. [3] Likert scale score reversed due to negatively stated item. [4] Contained the 12 Likert scale items with 2 additional closed-ended Likert scale items. [5] Contained the 12 Likert scale items along with 2 closed-ended Likert scale items from post-training survey, 1 additional closed-ended Likert scale item and an open-ended question.
