# Supplementary figures and images for: mHealth-Supported Hearing Health Training for Early Childhood Development Practitioners: An Intervention Study
Source: Int J Environ Res Public Health. 2022 Oct 31;19(21):14228. doi: 10.3390/ijerph192114228 (PMC9658621; doi:10.3390/ijerph192114228)

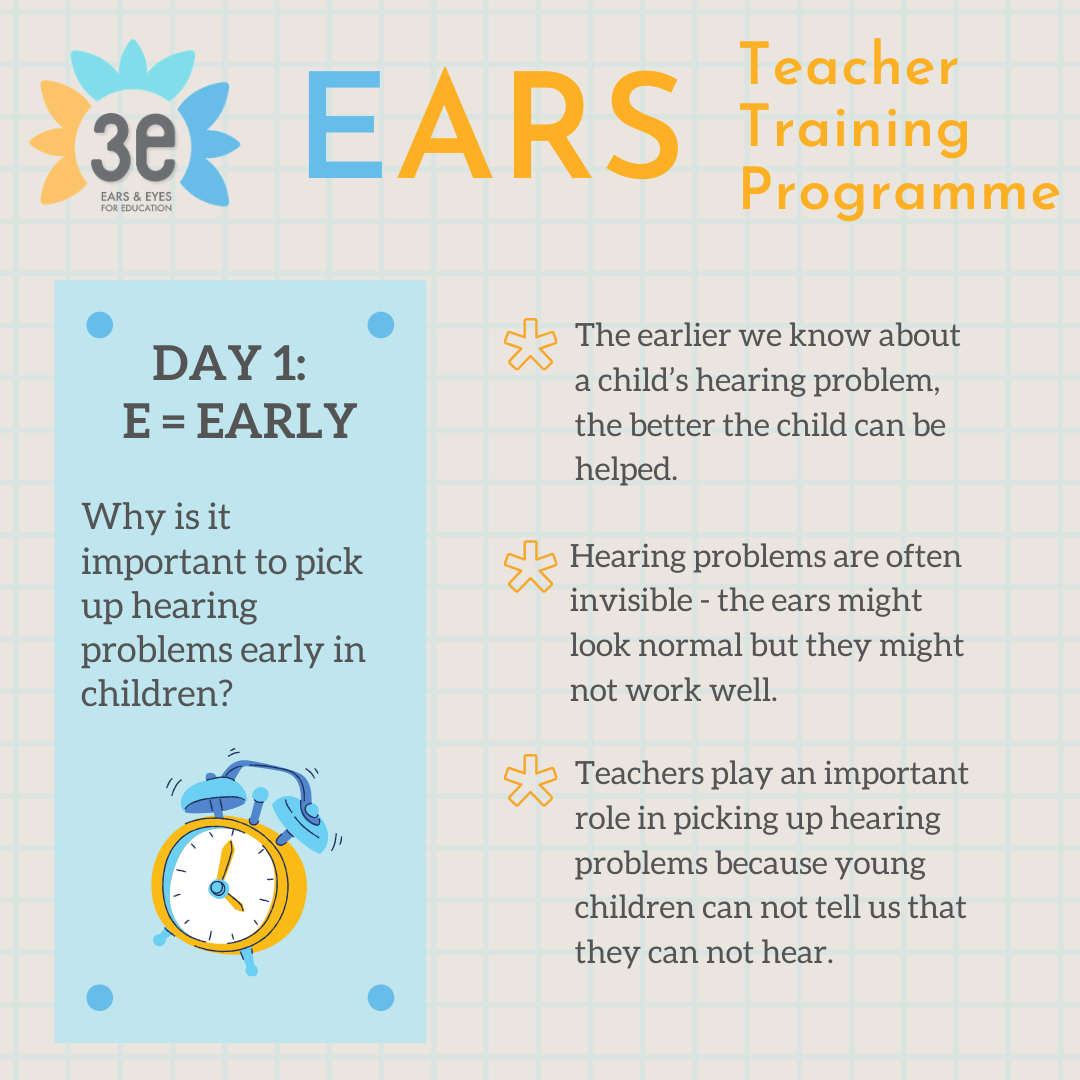

Supplement: Supplementary file 1 [file ijerph-19-14228-s001.zip › Multimedia Supplementary S2/EARS Training_Day1.png]

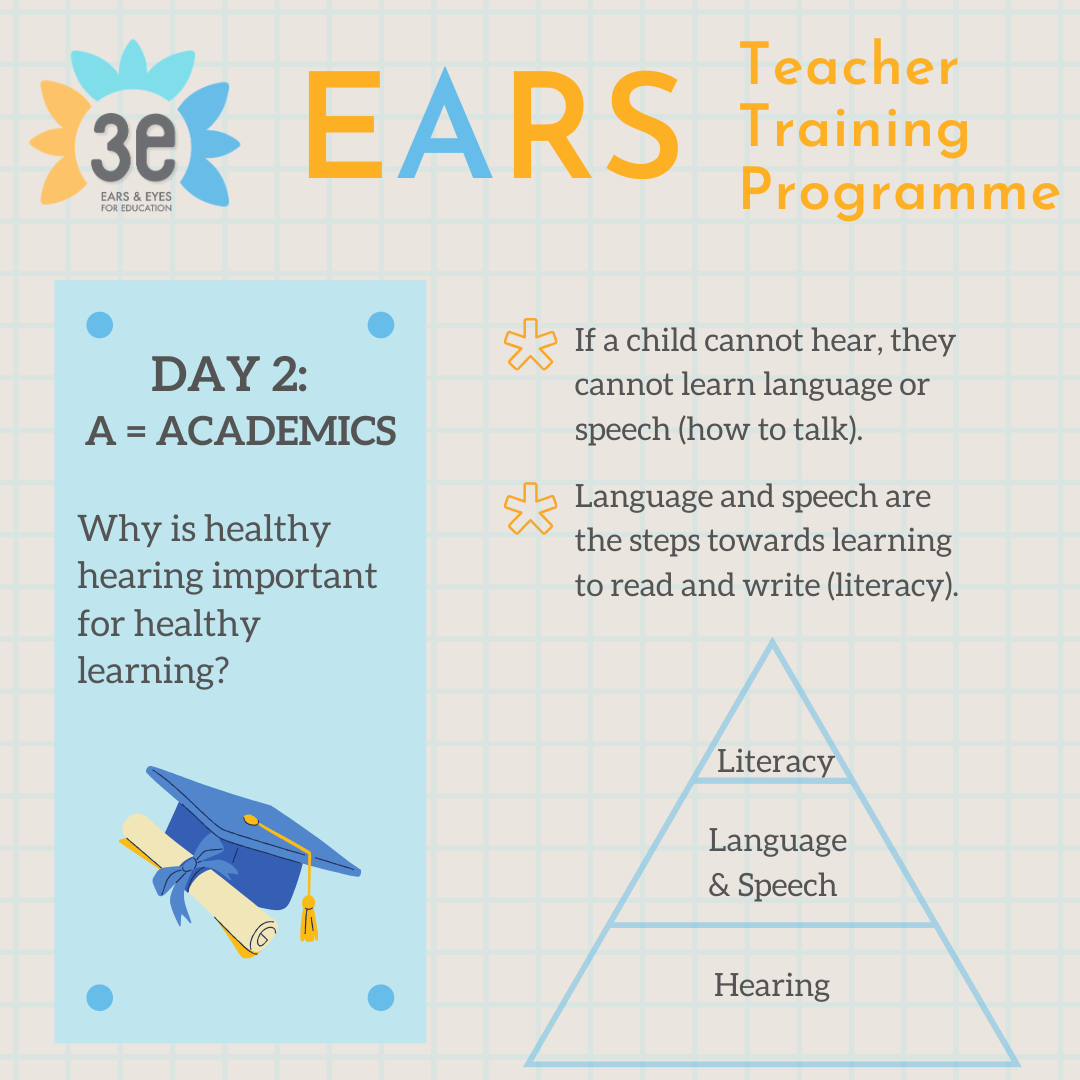

Supplement: Supplementary file 1 [file ijerph-19-14228-s001.zip › Multimedia Supplementary S2/EARS Training_Day2.png]

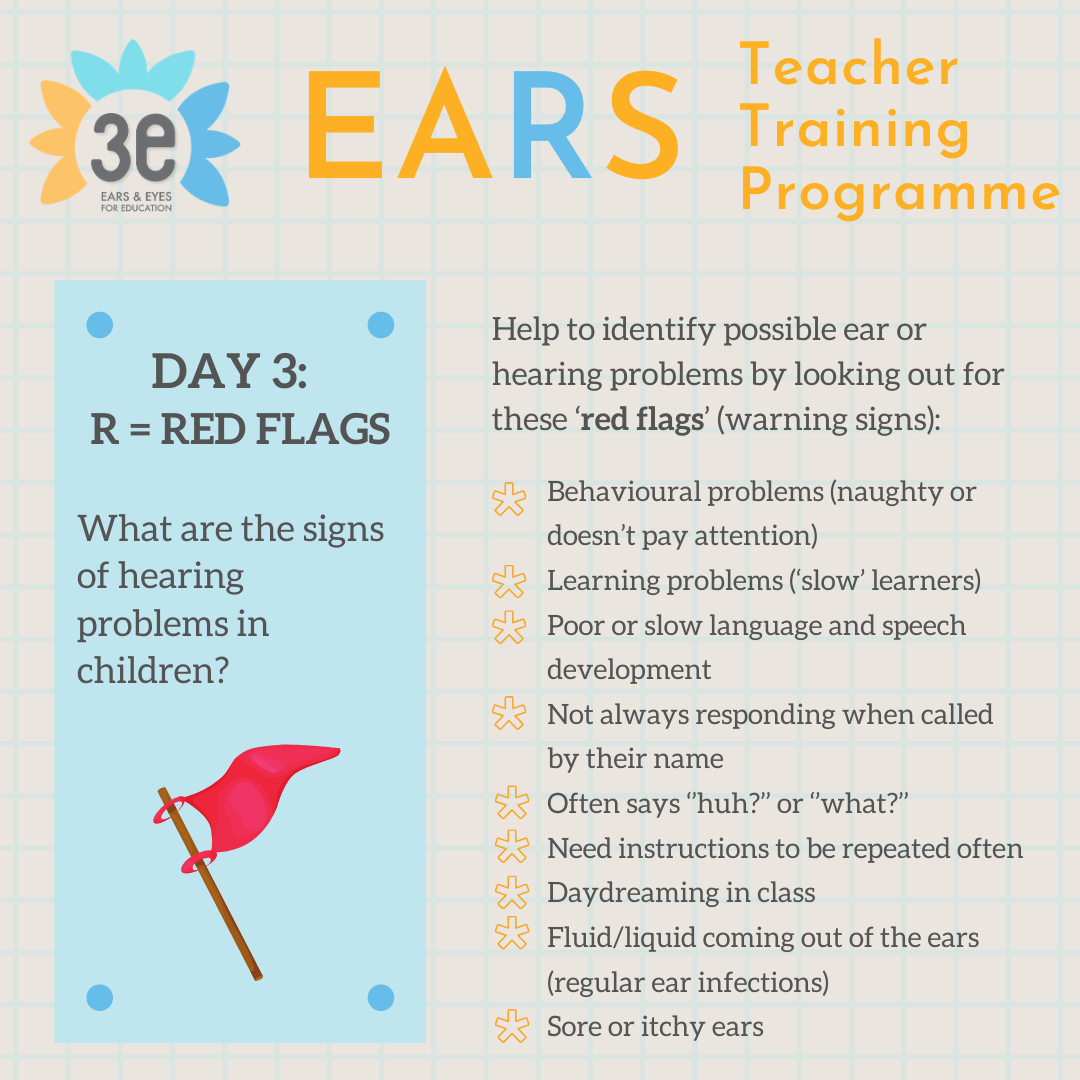

Supplement: Supplementary file 1 [file ijerph-19-14228-s001.zip › Multimedia Supplementary S2/EARS Training_Day3.png]

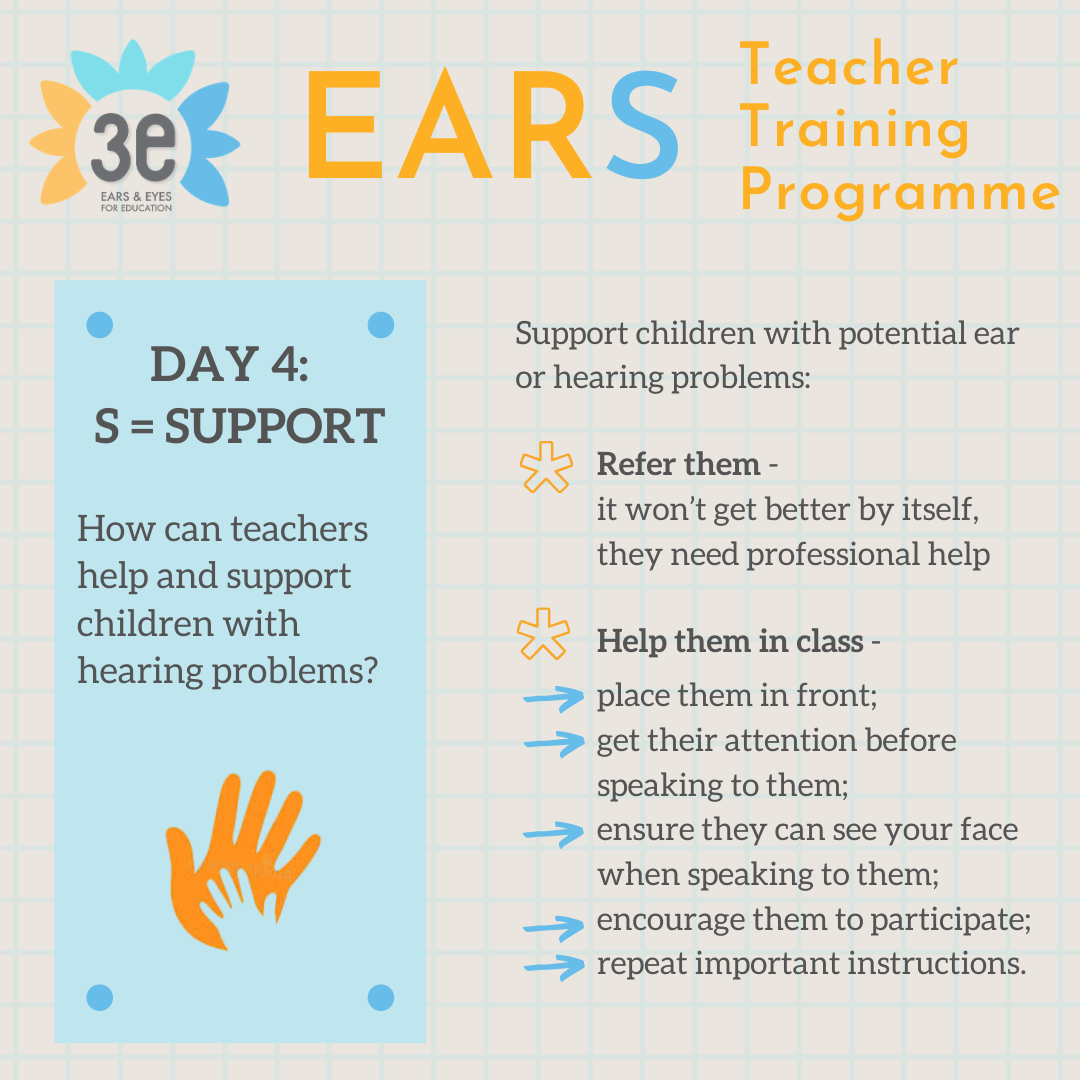

Supplement: Supplementary file 1 [file ijerph-19-14228-s001.zip › Multimedia Supplementary S2/EARS Training_Day4.png]
